# Design and Synthesis of Dipeptidomimetic Isocyanonaphthalene as Enhanced-Fluorescent Chemodosimeter for Sensing Mercury Ion and Living Cells

**DOI:** 10.3389/fchem.2022.813108

**Published:** 2022-03-04

**Authors:** Xiao-Juan Wang, Gao-Wei Li, Yi-Peng Cheng, Qiu-Ling Sun, Yuan-Qiang Hao, Chen-Hong Wang, Lan-Tao Liu

**Affiliations:** College of Chemistry and Chemical Engineering, Henan Engineering Laboratory of Green Synthesis for Pharmaceuticals, and Henan Key Laboratory of Biomolecular Recognition and Sensing, Shangqiu Normal University, Shangqiu, China

**Keywords:** dipeptidomimetic, isocyanonaphthalene, mercury ion, fluorescent probe, cell imaging

## Abstract

A novel valine-based isocyanonaphthalene (**NpI**) was designed and synthesized by using an easy method and enabled the selective fluorescence detection of Hg^2+^. The chemodosimeter can display an immediate turn-on fluorescence response (500-fold) towards target metal ions upon the Hg^2+^-mediated conversion of isocyano to amino within **NpI**. Based on this specific reaction, the fluorescence-enhancement probe revealed a high sensitivity toward Hg^2+^ over other common metal ions and exhibited excellent aqueous solubility, good antijamming capability, high sensitivity (detection limit: 14.2 nM), and real-time detection. The response mechanism of **NpI** was supported by NMR spectroscopy, MS analysis and DFT theoretical calculation using various techniques. Moreover, a dipeptidomimetic **NpI** probe was successfully applied to visualize intracellular Hg^2+^ in living cells and monitor Hg^2+^ in real water samples with good recoveries and small relative standard deviations.

**GRAPHICAL ABSTRACT:**
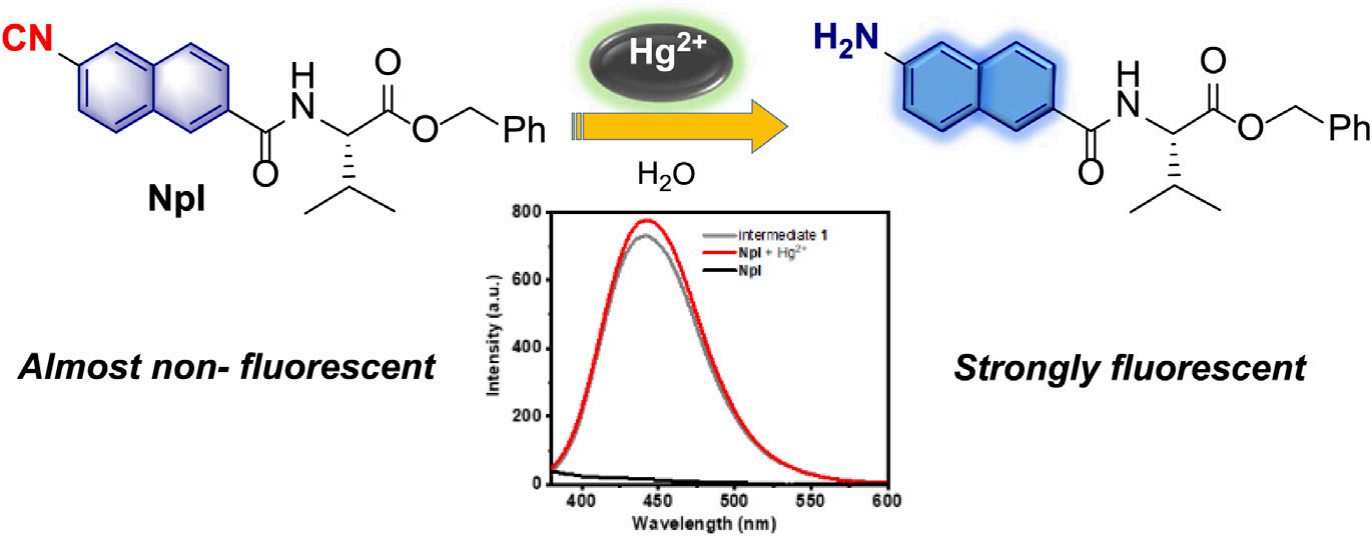


## Introduction

Mercury in the environment is one of the most hazardous heavy metals because of its known toxicity and accumulated features towards some aquatic life and humans. Various processes can lead to the release and accumulation of mercury from human activities, such as the burning of coal and gasoline, solid waste incineration, the daily diet of fish and sea mammals, and the instrument industry. Studies revealed that exposure of the body to inorganic mercury in health risks (mostly in the form of Hg^2+^), will result in substantial damage to the nervous system and endocrine system. Besides, these mercuric salts have a high affinity for thiol groups containing proteins and enzymes, which are mainly damaged kidneys and then widely distributed throughout the body. The quick and effective detection of Hg^2+^ analytics from possible natural and tap water pollution is of great importance (Celo, et al., 2006; Driscoll, et al., 2013). Therefore, developing novel fluorescent sensors for Hg^2+^ with good selectivity, high sensitivity. and more importantly applicable to complex environmental and biological systems, has received considerable interest in the areas (Carter, et al., 2014; Wu, et al., 2017).

Over the past few years, researchers have tried to develop efficient Hg^2+^ probes based on small organic molecules, polymers, and metal-organic complexes (Chen, et al., 2019; Lu, et al., 2016; Oing, et al., 2021; Ru, et al., 2014; Singha, et al., 2019; Thomas, et al., 2007). Fluorescent sensors based on small molecules, in particular, have significant advantages such as their exquisite and noninvasive size, high sensitivity, selectivity, and fast response time. To date, numerous fluorescent Hg^2+^ (Chen, et al., 2015; Kim, et al., 2012) probes have been reported based on varieties of fluorophore like Rhodamine (Bera, et al., 2014; Chang, et al., 2018; Su, et al., 2017; Tang, et al., 2017; Zhao, et al., 2010), BODIPY (Hatai, et al., 2012; Lu, et al., 2009; Yoon, et al., 2005), Coumarin (Sie, et al., 2017; Puthiyedath and Bahulayan, 2018), Schiff base (Chen, et al., 2018; Duan, et al., 2019; Sarkar, et al., 2016; Wang, et al., 2018b; Wang, et al., 2018d; Zhang, et al., 2017), AIE luminophore (Chen, et al., 2017; Selvaraj, et al., 2021; Wang, et al., 2018a), Peptide-based benzoxazole unit (Oliveira, et al., 2011; Oliveira, et al., 2013), etc. These probes were usually prepared either through the complexation with the multiple binding sites or through a chemical reaction with the proper functional group onto fluorescent molecules. However, some of the sensors often show some limitations, such as laborious and expensive synthesis, fluorescence quenching measurement, poor aqueous solubility or slow dissolution, and limited selectivity as a result of interference from competing metal ions. Therefore, the development and improvement of specific responses towards Hg^2+^ based on fluorescence-enhancement, which leads to more sensitivity and robustness than fluorescence-quenching, is still a highly active field in environmental sensor and sensing.

Currently, fluorescent probes based on naphthalene (Np) skeleton have received much attention over the last several years (Banerjee, et al., 2012; Roek, et al., 2000). Among many classes of fluorophores, Np provides an ideal platform that can be derivatized with ease for design and development of fluorescent chemosensors such as, fluorescent sensors derived using naphthalene moiety and suitable amine derivative which often provide an excellent functionalized backbone for a variety of analytes (Das and Goswami, 2017), including the hydrazide derivative containing naphthyl backbone (Pannipara, et al., 2018), various naphthyl Schiff base derivatives (Das, et al., 2013; Zhang, et al., 2013), naphthalimides (Shu, et al., 2015) and naphthyl polymer materials (DeRosa, et al., 2014).

In addition, the isocyano group of isocyanides is an extremely versatile and functional building block with excellent reactivity including the ability to react with both nucleophiles, electrophiles, and radicals, simultaneously (Songab and Xu, 2017; Zhang and Studer, 2015; Zhang, et al., 2020). They are used as privileged synthons for multicomponent reactions (MCRs), heterocyclic ring formation, and other insertion reactions in organic and drug synthesis (Chakrabarty, et al., 2014; Sadjadi, et al., 2016; Vavsari, et al., 2020). Isocyanides have also attracted the attention of coordination chemists due to their strong σ-donor and weak π-acceptor properties (Boyarskiy, et al., 2015; Yamamoto, 1980), and for advances in the synthesis and application of helical polymers, and the screw-sense polyisocyanides have been extensively used to the living polymerization of the corresponding isocyanide monomers (Wang, et al., 2018c; Zhou, et al., 2021). Notably, the chemical reactions based on the isonitrile group have recently been exploited for the design of fluorescent Hg^2+^ probes (Adamoczky, et al., 2020; Nagy, et al., 2019; Zhang, et al., 2018), which involves the Hg^2+^-mediated conversion of isonitrile to the amino group. Only two isocyano-functionalized fluorescent probes thay display distinct optical response have been explored. Xie group synthesized a isocyano-functionalized 1,8-naphthalimide derivative for ratiometric fluorescent sensing of Hg^2+^. Subsequently, the Kéki group designed a series of isocyanonaphthalene (NpI) derivatives as fluorescence probes for Hg^2+^. However, some problems still exist such as the use of substantial amounts of organic solvent, the relatively slow response time, and poor anti-interference ability.

**SCHEME 1:**
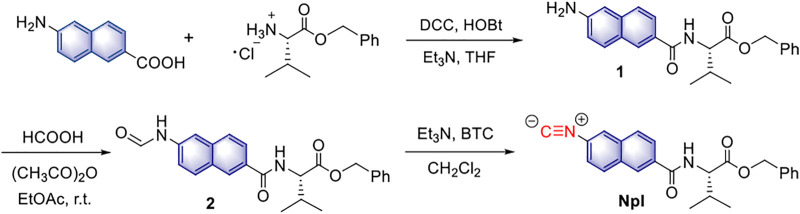
The synthesis of sensor **NpI**.

Recently, we developed a series of new polyisocyanopeptides bearing amino-acid ester side chains as a novel weakly orienting medium for RDC-based configurational analysis of small organic molecules, and the related dipeptidomimetic isonitrile monomers of polyisocyanides are much synthesized and studied (Li, et al., 2017; Li, et al., 2018). To our best knowledge, compared with the well-established study of isocyanides in polymerization and organometallic domain, and traditional organic synthesis, the construction of dipeptidomimetic isocyanide-based chemosensors was rarely focused on, which may be due to its high reactivity and the lack of excellent synthesis methods. Furthermore, the introduction of amino-based dipeptidomimetic pendant to chromophores can efficiently increase its biocompatibility for fluorescent bioimaging in living systems. Herein, we disclose a novel valine-based naphthyl isocyanide (named **NpI**, Scheme 1) for trace level detection of mercury ions and visualize its bio-imaging in live cells. The enhanced fluorescent probe exhibited distinctive selectivity over other metal ions, fast response, and excellent sensitivity toward Hg^2+^. In this work, we present its synthesis depending on our mature method, characterization, sensing behavior in detail, and the related mechanism which was also described by the reduction of the isonitrile group to the resulting primary amine as a chemical reaction process. Furthermore, valine-based dipeptidomimetic isocyanonaphthalene chemosensor **NpI** can be successfully applied to the imaging of intracellular Hg^2+^ ions due to its good biocompatibility.

## Materials and Methods

### Materials

Dimethyl sulfoxide (DMSO), analytical HgCl_2_, all synthetic starting materials, and solvents were used as received without further purification. Dry dichloromethane (DCM) was freshly distilled over calcium hydride (CaH_2_) under argon atmosphere. The chlorate salts used in the form of aqueous stock solutions were Al^3+^, Co^2+^, Cd^2+^, Cr^3+^, Fe^3+^, Ca^2+^, K^+^, Mg^2+^, Li^+^ and Na^+^, and other Cu^2+^, Mn^2+^, Ni^2+^, Pb^2+^, Sr^2+^ and Zn^2+^ were prepared from its sulfate salts. These stock solutions were provided by the Key Laboratory of Biomolecular Recognition and Sensing of Shangqiu Normal University. All fluorescence measurements were performed at room temperature on Agilent technologies carrying an eclipse fluorescence spectrophotometer. UV-visible spectra were recorded on a Perkin-Elmer (Lambda 850) spectrometer. The pH measurements were made with a Model PHS-3C. The measurement procedure including the stock solution of **NpI**, the stock solution of analytes, etc., are presented in detail in the Supplementary Material.

### Synthesis and Characterization of Mercury Sensor NpI

The synthesis of intermediates 1-2 was described in detail in the Supplementary Material. Under the argon atmosphere, *N*-formyl-dipeptidyl amide 2 (0.85 g, 2.1 mmol) was dissolved in 25 ml dry CH_2_Cl_2_, triethylamine (Et_3_N, 0.75 ml, 5.5 mmol, 2.0 equiv) was added, and the reaction mixture was cooled to 0°C. Over a period of 1 h, a solution of triphosgene (BTC, 443 mg, 1.5 mmol) in 10 ml dry CH_2_Cl_2_ was added dropwise into the mixture *via* additional vessel, the temperature of reaction system was maintained at 0°C. The resulting reaction mixture was stirred for an additional 3 h at room temperature. An ice-cold saturated NaHCO_3_ aqueous solution (10 ml) was added and the mixture was stirred vigorously for 5 min. The organic part was separated and next extracted with CH_2_Cl_2_, washing once with brine and dried over anhydrous Na_2_SO_4_, filtered, and concentrated. Purification on silica column chromatography (Petroleum ether: EtOAc = 3:1) afforded desired probe **NpI** (0.61 g, yield: 81%). M. p. = 128.1–128.5°C. [α] ^20^_D_ = +47.48 (*c* 0.20, in CHCl_3_). ^1^H NMR (400 MHz, CDCl_3_) *δ* (ppm) 8.30 (d, *J* = 1.7 Hz, 1H), 7.94–7.86 (m, 4H), 7.47 (d, *J* = 8.7 Hz, 1H), 7.43–7.30 (m, 5H), 6.87 (d, *J* = 8.6 Hz, 1H), 5.27 (d, *J* = 12.2 Hz, 1H), 5.20 (d, *J* = 12.2 Hz, 1H), 4.89 (dd, *J* = 8.6, 4.8 Hz, 1H), 2.37–2.32 (m, 1H), 1.02 (d, *J* = 6.9 Hz, 3H), 0.98 (d, *J* = 6.8 Hz, 3H). ^13^C NMR (100 MHz, CDCl_3_) *δ* (ppm) 172.1, 166.7, 165.5, 135.2, 134.2, 133.2, 132.1, 130.8, 128.8, 128.7, 128.6, 128.5, 128.3, 127.5, 125.6, 125.4, 124.4, 67.4, 57.6, 31.8, 19.1, and 17.9. FT-IR (cm^−1^): 3265, 2950, 2125, 1730, 1635, 1535, 1200, 700. HRMS (ESI): m/z: anal. calcd for C_24_H_22_N_2_O_3_ [M + Na]+409.1630, found 409.1649.

## Results and Discussion

### Design and Synthesis of Probe NpI

The **NpI** is composed of a naphthalene fluorophore bearing a valine benzyl ester as the pendant and a -N≡C moiety as a specific recognition site for Hg^2+^. The practical synthetic route of the new dipeptidomimetic **NpI** is shown in Scheme 1, and the target compound and related precursors were fully characterized *via* standard NMR spectroscopy and high-resolution mass spectrometry (HRMS).

A concise and highly efficient synthetic route to dipeptidomimetic **NpI** is presented here. The traditional method of preparing the intermediate **1** analogue is cumbersome and not environmentally friendly. Such as, intermediate **1** has been prepared mainly by condensation reaction with *para*-nitro aromatic acid, and then a need for reducing the nitro group of the aromatic moiety under Pd/C catalyzed hydrogenation condition, and the synthesis of this compound usually consists of two steps (Kajitani, et al., 2006; Reller, et al., 2017). Herein, a novel method is proposed to generate dipeptidyl amide **1** from the corresponding *para*-amino aromatic acid instead of *para*-nitro aromatic acid. Thus, the synthesis of sensor **NpI** is very straightforward through a three-step synthetic route (Scheme 1). First, the direct coupling reaction of 6-amino-2-naphthoic acid and valine benzyl ester affords dipeptidyl amide **1** in 84% yield. *N*-Formylation of intermediate **1** with formic acid proceeds smoothly to afford the desired intermediate **2** in 72% yield. Finally, base-promoted conversion of triphosgene with **2** *via* a dehydration reaction furnishes **NpI** in 81% yield (the general synthesis procedure is illustrated in Supplementary Scheme S1).

As referred to previously, the synthesis of NpI bearing valine benzyl ester is crucial in fluorescent sensor applications. Therefore, the chemical structure of the novel dipeptidomimetic **NpI** has been elucidated by means of spectroscopic methods. In FT-IR spectrum of **NpI**, the characteristic vibration for the ester carbonyl C=O bond and amide carbonyl C=O bond was located at 1730 and 1635 cm^−1^ respectively, and the stretching vibration absorption of isocyanic N≡C bond were clearly observed at 2125 cm^−1^. ^1^H NMR spectrum of **NpI** showed to two doublet signals at *δ* ∼1.0 and multiple signals at ∼2.35 ppm, which corresponds to the classical isopropyl group of valine residue (Supplementary Figure S5), suggesting that the valine-based dipeptidomimetic has been completely condensed onto naphthalene backbone. The successful synthesis of sensor **NpI** can also be supported by ^13^C NMR structural characterization (Supplementary Figure S6). The carbon signal of **NpI** exhibits ester- and amide-carbonyl carbon signals at *δ* ∼172 and ∼166 ppm, significantly, and a quaternary carbon of isocyano group showed to the lowest signal at *δ* ∼165 ppm. According to the above-mentioned detailed characterization analyses, we can conclude that the novel dipeptidomimetic **NpI** sensor has been successfully synthesized in three steps using a simple methodology.

### Absorption Spectroscopy of Dipeptidomimetic NpI to Hg^2+^

**FIGURE 1:**
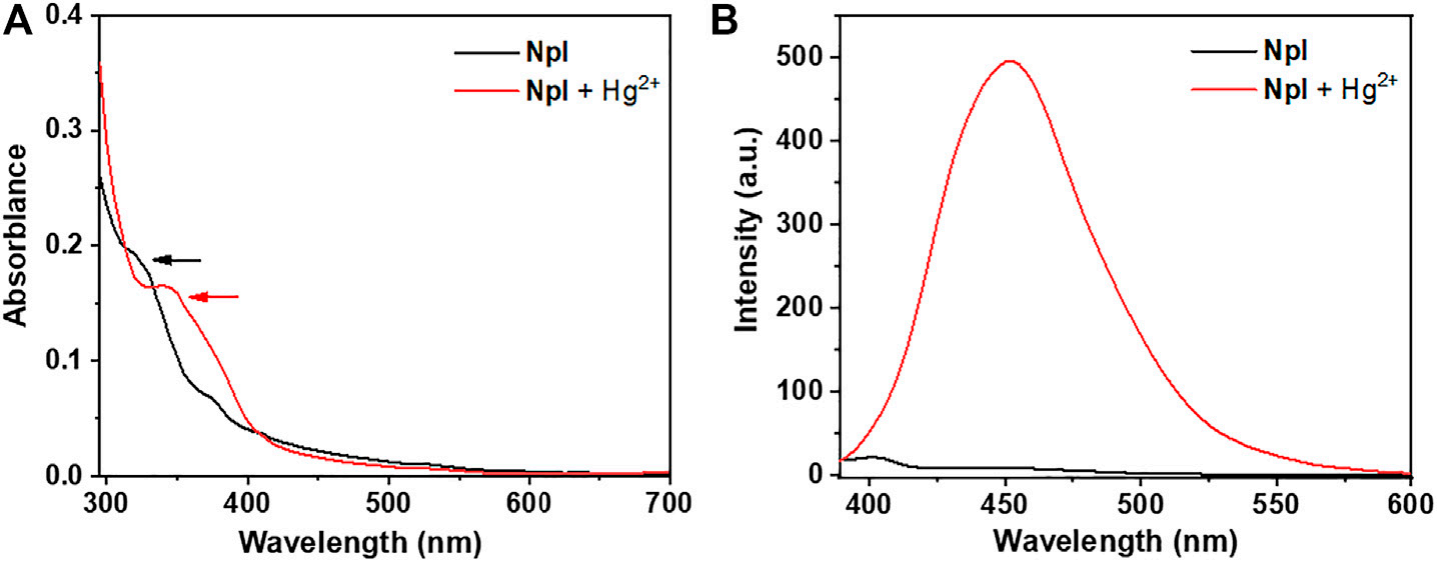
**(A)** Absorption spectra of **NpI** (black line) and **NpI** + 1.0 equiv HgCl_2_ (red line) in PBS buffer solutions. **(B)** Emission spectra of **NpI** (black line) and in the presence of 1.0 equiv HgCl_2_ (red line) in PBS buffer solutions.

With probe **NpI** on hand, the spectral properties of the sensor in the presence or absence of Hg^2+^ ions were determined. We first checked the UV-vis spectrum of the probe, as shown in Figure 1, and the absorption spectrum of dipeptidomimetic **NpI** has exhibited an obvious maximum absorption peak around 320 nm in the PBS buffer solutions at room temperature (containing 0.5% DMSO), which can be ascribed to the naphthyl ring π-scaffold transition band. Whereas, a new broad absorption band centered at around 350 nm was monitored on incremental addition of Hg^2+^, which is slightly red-shifted from the absorption maximum in solution. Consequently, fluorescence spectra were recorded under UV excitation, the free **NpI** has almost non-fluoresce emissive. However, the fluorescent intensity of **NpI** increased rapidly upon the addition of Hg^2+^, and 1.0 equitv of Hg^2+^ ions triggered a 500-fold emission enhancement and the fluorescence behavior was similar to that of naphthaline fluorophore (Zhang, et al., 2018). Then, the influence of the buffer system on the fluorescence property and response of probe **NpI** to Hg^2+^ was investigated. The screening of solutions indicates that the PBS buffer solutions containing 0.5% DMSO were optimal and suitable for further sensing application (Supplementary Figure S10).

### Selectivity Investigation and Fluorescence Titration Investigation

**FIGURE 2:**
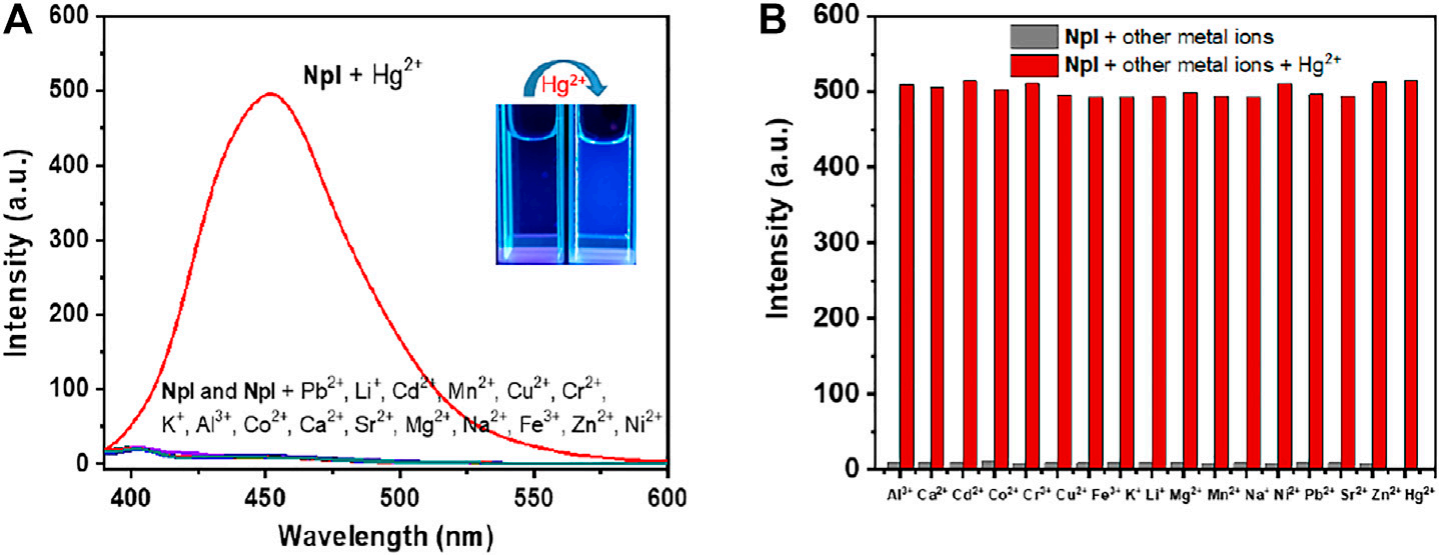
**(A)** Fluorescence enhancement of NpI (1 μM, pH = 7.4, 10 mM PBS) in the presence of other competing metal ions (1 μM, *λ*_ex_ = 350 nm). Inset: fluorescence changes of NpI under the portable UV lamp at 365 nm by the naked eyes. **(B)** Column diagram for fluorescence intensity changes of probe NpI (1 μM) to various 1 μM competing transition-metal ions. The lower one (gray bar) is in the presence of only competing metal ions with NpI; the higher one (red bar) is in the presence of a mixture of the analyte and Hg^2+^ with NpI.

To obtain an insight into the probing property and demonstrate the high selectivity of probe NpI in practice, different metal cations were introduced into the solution of NpI, and we investigated the effects of 17 different metal ions on the fluorescence of NpI under the same conditions. An enhancing effect was observed while interacting with Hg^2+^, while the individual addition of other ions like Al^3+^, Ca^2+^, Cd^2+^, Ni^2+^, Co^2+^, Cr^3+^, Cu^2+^, Fe^3+^, K^+^, Li^+^, Na^+^, Mg^2+^, Mn^2+^, Pb^2+^, Sr^2+^and Zn^2+^ exerted no effect on any apparent emission enhancement. Figure 2 depicted the fluorescence responses of NpI to various environmentally relevant metal ions. These observations indicate that probe NpI only showed sensitively fluorescent turn-on response to Hg^2+^ ions. Interestingly, from the photos of probe NpI before and after treatment of Hg^2+^, we can clearly find that with the addition of Hg^2+^, the UV light probing platform displayed weak alteration (under portable 365 nm UV lamp), it emitted visible pale-blue light, and such alteration can be easily observed by the naked eyes. The finding results suggest that NpI has the immense potential to act as a selective Hg^2+^-detection probe. Moreover, to further confirm the selectivity of the tested metal sensing response of NpI, the competitive experiments were subsequently carried out. The experimental results were presented in Figure 2, as observed, the fluorescence emission intensity monitored in the presence of 1.0 equiv of Hg^2+^ ion mixed with 1.0 equiv of various cations, respectively, red columns represented the fluorescence intensity of probe NpI in the presence of Hg^2+^ and other competing metal ions. These aforementioned results indicated that NpI was not interfered with by other metal cations and a Hg^2+^ ion-specific fluorescent recognition sensor. Such an outstanding specificity would effectively avoid false positives.

**FIGURE 3:**
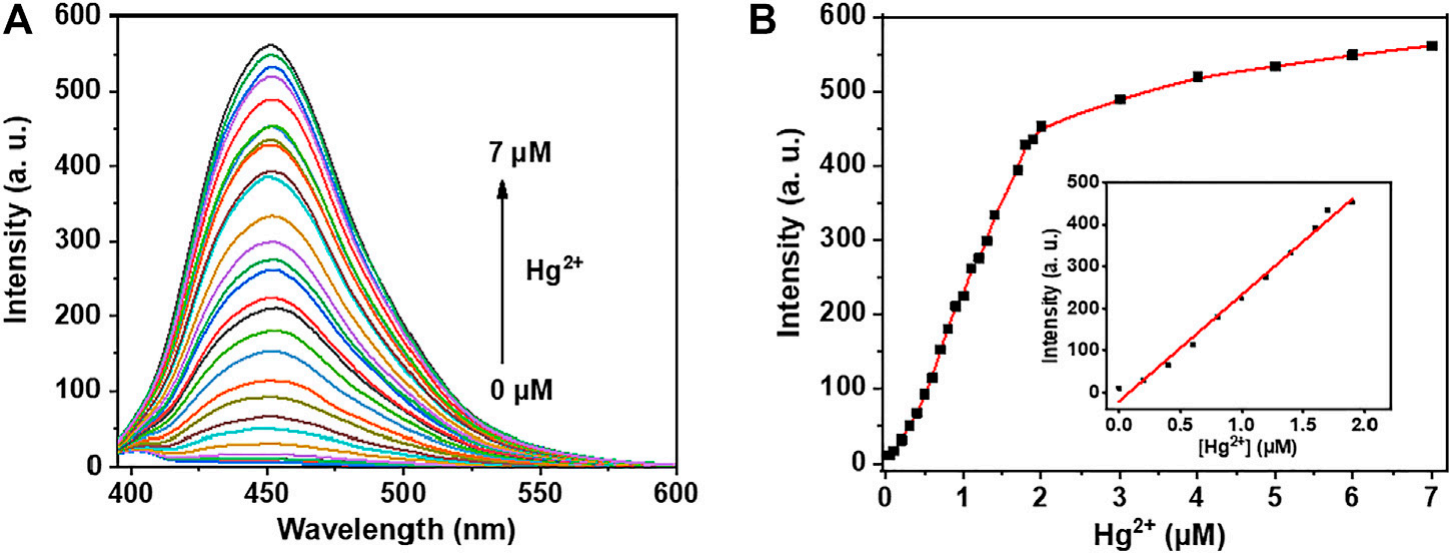
**(A)** Fluorescence titration of **NpI** (1 μM) with Hg^2+^ ion (0–7.0 μM) in PBS aqueous buffer (10 mM, pH = 7.4). **(B)** Linear relationship between fluorescence emission intensity and added Hg^2+^ concentrations. Inset: the calibration curve, the linear relationship is expressed as y = 253x−19.9 (*R* ^2^ = 0.991).

Under the optimized condition, the sensitivity of probe **NpI** was then carried out by fluorescence emission response toward different concentrations of Hg^2+^ ion, as shown in Figure 3. As expected, the fluorescence intensity increased upon incremental addition concentrations of Hg^2+^. While the fluorescence intensity enhanced nearly 500-fold when 1.0 equiv Hg^2+^ ion was added to the same solution. The plot of fluorescence intensity of **NpI** at 450 nm linearly increased up to gradual addition of Hg^2+^ concentration from 0.1 to 2.0 μM. There is a good linear relationship between the fluorescence response and the added Hg^2+^ concentration (*R* ^2^ = 0.991). The detection limit of **NpI** for the concentration-dependent response manner of Hg^2+^ was measured to be 14.2 nM (2.8 ppb) based on 3σ/k.

### The Effect of pH and Responding Speed Investigation on the Detection of Hg^2+^

**FIGURE 4:**
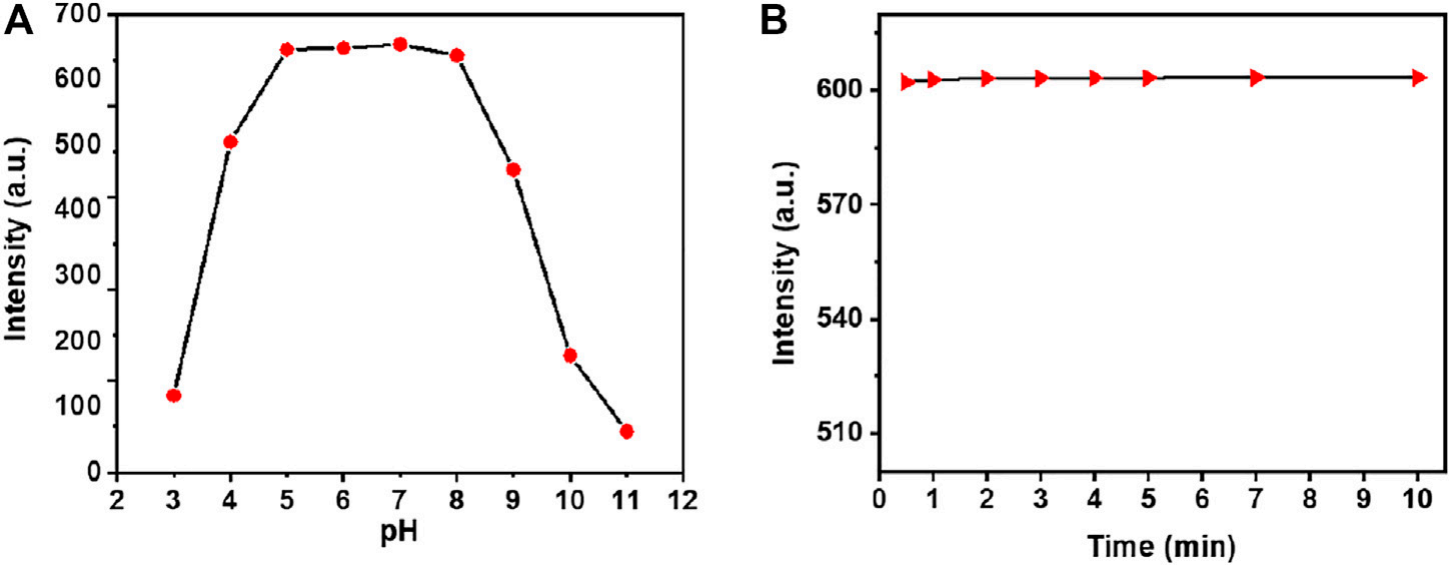
**(A)** The pH titration plots for probe NpI at different pH PBS buffer solutions (containing 0.5% DMSO as a cosolvent). **(B)** Time evolution of NpI (1 μM) in PBS aqueous (pH = 7.4) in the presence of 1.0 equiv Hg^2+^ ion.

The detection of heavy and transitional metal ions in acidic conditions is indispensable for practical applications because the low pH facilitates metal solubility in water, and the contamination of heavy ions to the environment system, will be more serious in acidic conditions, and it would be more useful in identifying the total soluble heavy metal ions in usual water. Therefore, studies on the pH effect (in the range of pH 3.0–11.0 of sensing PBS buffer) on the fluorescence response of NpI were observed in the presence of Hg^2+^ ion. As recorded by the results in Figure 4, the probe NpI was stable and provided a good spectral response to HgCl_2_ heavy metal ion over a wide pH range from 5.0 to 9.0, indicating that the probe can endure the acidity of the river and lake water and has a strong spectral response to the analyte (Supplementary Figure S11). As a chemosensor for practical applications, short responding time or high response speed is advantageous for sensors to raise the detection efficiency in a certain range, that is, realize the real-time analysis. Therefore, the time-dependent fluorescent changes of the NpI (1 μM) in the presence of 1.0 equiv of Hg^2+^ ion was measured under the same PBS buffer solution. As can be seen from Figure 4, the recognition interaction was almost completed within 1 min, and it showed an almost undetectable time-delay when the addition of Hg^2+^ ion. The result showed that sensor NpI was a sensitive sensor and could be applied in real-time monitoring of target metals in some environmental analysis.

### Plausible Detection Mechanism and Theoretical Computations

**FIGURE 5:**
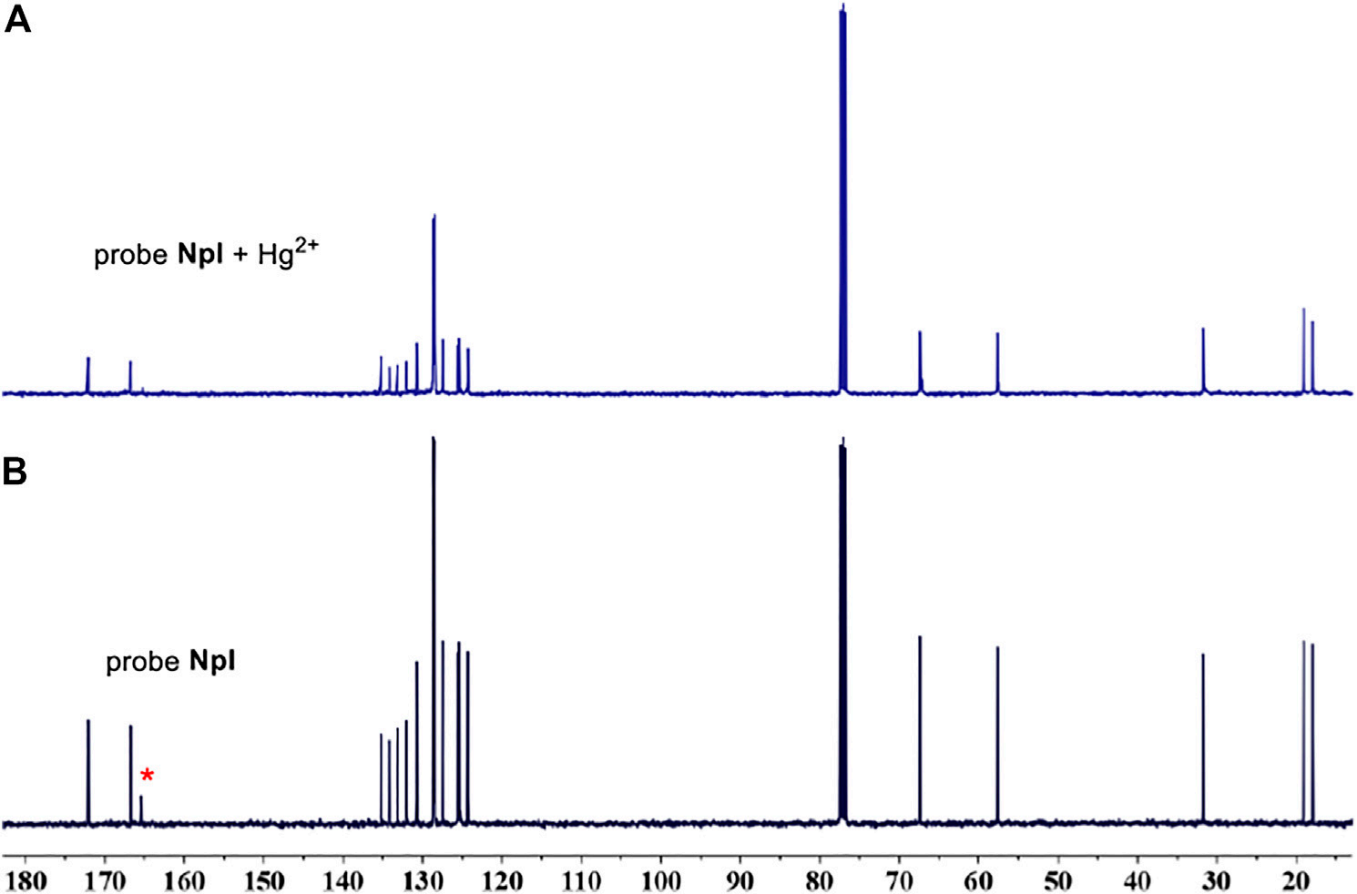
**(A)** The ^13^C NMR (CDCl_3_, 400 MHz, 298 K) spectra of NpI. **(B)** Evolution of the ^13^C NMR spectra of NpI with the addition of HgCl_2_.

According to similar mechanism studies (Adamoczky, et al., 2020; Nagy, et al., 2019; Zhang, et al., 2018) which involve aromatic isocyanides as high effective sensors for the quantification of target metal Hg^2+^, the sensing mechanism can be also speculated to the Hg^2+^-induced hydration of isocyano group to amino group in water. To verify this conversion process, the fluorescence response of precursor compound, i.e., intermediate **1,** was firstly investigated toward Hg^2+^ under the identical measurement experimental condition of dipeptidomimetic **NpI** sensor. One can find that the precursor intermediate **1** was an almost indiscernible response to Hg^2+^ (Supplementary Figure S12). Meanwhile, the fluorescence spectra of **NpI** + Hg^2+^ and intermediate **1** were almost the same to each other, and this phenomenon has been brought to our attention. To get circumstantial evidence regarding the probing detection mechanism, NMR experiments have been performed by the concomitant addition of **NpI** into HgCl_2_ in deuterated reagents. Figure 5 shows the ^13^C NMR spectrum of free **NpI** in CDCl_3_, the ester-, amide-carbonyl, and isocyanic carbon signals were clearly discernible at *δ* 172, 166, and 165 ppm, respectively. However, upon the addition of a certain amount of HgCl_2_, the signal intensity of the isocyano group gradually disappeared at *δ* 165 ppm. The above results indicated the conversion of the isocyano moiety to amino group in water by Hg^2+^-assisted hydration reaction. Subsequently, we carried out experimental verification by quickly mixing **NpI**/HgCl_2_ in chloroform system, the reaction solution immediately turned yellow-ish green, the mixture was washed with H_2_O and purified on TLC, and the **NpI**/Hg^2+^-induced trace product was analyzed by ^1^H NMR. Interestingly, the spectra of **NpI**/Hg^2+^and intermediate **1** were exactly the same (Supplementary Figure S13), suggesting that the NpI-based probing system could be, indeed, assigned to reaction-type probing mechanism instead of complexation mechanism.

To further get direct evidence for reaction-type products formed in the PBS aqueous solution/DMSO mixture, ESI-MS analysis was performed from aqueous medium. The 1:1 reaction mixtures were immediately analyzed similarly to those for the fluorescence experiments by HRMS (Supplementary Figure S14). The determination of mass spectrum from **NpI**/Hg^2+^-induced trace product displayed at m/z of 377.1962, which was consistent with the intermediate **1** (m/z calc = 376.18). Based on above analyses, the plausible probe mechanism was described by the reduction of the isonitrile moiety to primary amine as a chemical reaction-type process.

**FIGURE 6:**
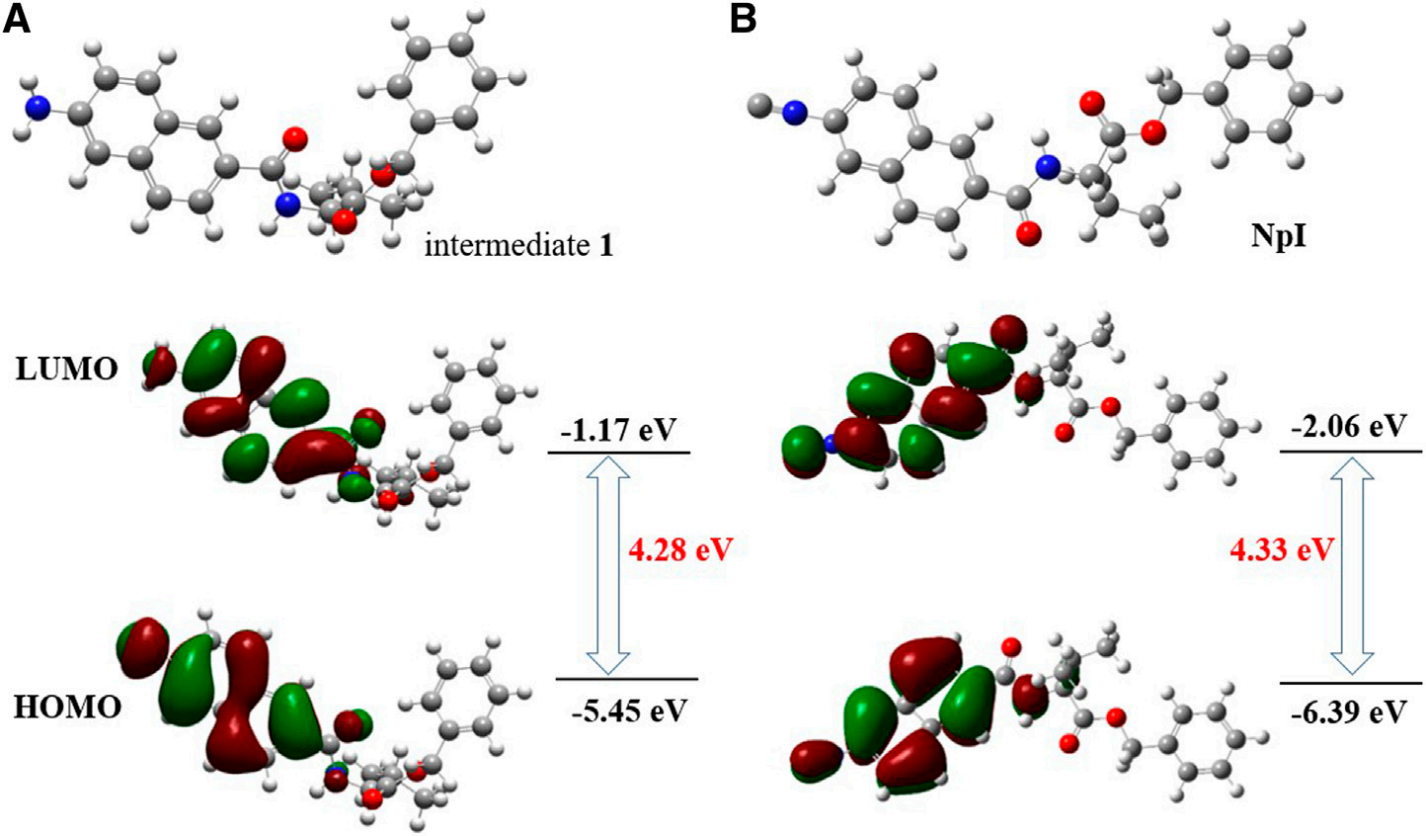
Calculated DFT energy and frontier molecular orbital diagrams of **(A)** precursor 1 and **(B)** NpI.

DFT theoretical calculations were also performed to further elucidate the above experimental findings, the optimized electronic structural models of precursor **1,** and **NpI** calculated at B3LYP/6-31G (d,p) level of theory using Gaussian16. Calculated electron density surfaces of HOMO/LUMO of intermediate **1** and **NpI** are displayed in Figure 6. As revealed by relative energies and electron density, HOMO electron of precursor **1** focused on the amino and naphthalene core, and the marked conjugation bridge was established between two phenyl groups of naphthalene units, LUMO electron of precursor **1** distributed mainly in the scaffold of naphthalene, which can lead to a smaller energy gap between HOMO and LUMO (4.28 eV). This theoretically supports that the intramolecular charge transfer (ICT) process had tremendous importance due to the electron-donating amino group. However, as for **NpI**, the ICT effect seems much weaker and the electron bridge was not observed in its HOMO. Our DFT calculations reveal that the larger HOMO-LUMO energy gap was formed as the result of poor electronic distribution due to the electron-withdrawing isocyano group.

### Applications in Living Cells and Real Water Samples

**FIGURE 7:**
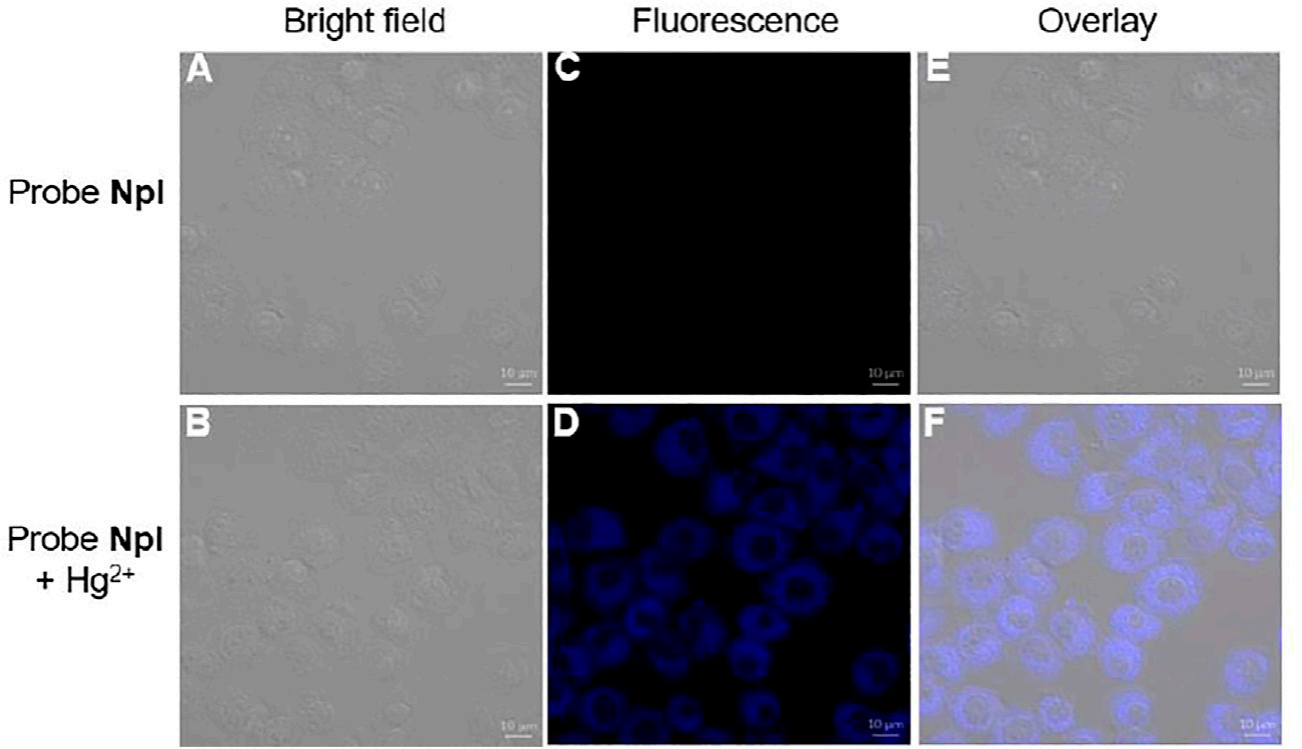
Fluorescent confocal bioimaging of MCF-7 cells: **(A, C, E)** cells incubated with free probe **NpI**, **(B, D, F)** images were captured after 30 min of treatment with probe **NpI** and Hg^2+^, respectively. **(A, B)** bright-field images, **(C, D)** fluorescence images, **(E, F)** merged images.

The good fluorescence property of NpI prompted us to check the potential application in a living system, in addition, NpI-containing valine residue as chemosensor may be applied to the imaging of intracellular Hg^2+^ due to its known biocompatibility. As shown in Figure 7, the bioimaging application of NpI to detect intracellular Hg^2+^ ion was observed by fluorescence confocal images of MCF-7 cells (human breast adenocarcinoma cell line). The MCF-7 cells were incubated with free probe NpI (10 µM) for 1 h and showed no detectable fluorescence. After further incubating NpI-stained MCF-7 cells with Hg^2+^ ion (50 µM) for another 1 h, a weak blue emission was initially noticed. These fluorescent imaging experimental results demonstrate that dipeptidomimetic NpI has the capability to visualize intracellular Hg^2+^ ion for biological samples.

The potential of probe NpI to detection of Hg^2+^ in real water samples were demonstrated, including tap water, river water (Shangqiu Yellow River Old Riverway) and lake water (Shangqiu South Lake). The collected water samples were filtered (0.22 mm filter) and the pH were adjusted to 7.4 using a phosphate buffer. Each pretreated sample was mixed with an equal volume of DMSO and then spiked with different concentrations of Hg^2+^. Then 10 μM of probe was added into each sample and the fluorescence spectrum was measured. As shown in Table 1, the calculated recoveries ranging from 93.9% to 105.8%, the relative standard deviations (RSDs) are less than 6.4%, and the relative error is less than 6.5%. The performance of our synthesized probe NpI identified for selective Hg^2+^ detection was also compared with some preciously reported fluorescent Hg^2+^ chemosensors (Supplementary Table S1). It could be seen that the proposed probe exhibits comparable or superior analytical performances in water as compared to some of earlier reported probes. These observations indicated that probe NpI can be potentially utilized in environmental samples.

**TABLE 1:** Application of probe **NpI** in determination of Hg^2+^ in actual samples.

Sample	Added (μM)	Detect (μM)	RSD (%)	Recovery (%)	Relative error (%)
River water	20	18.9	6.4	105.8	6.0
	40	41.6	4.1	96.2	4.0
	80	79.7	1.3	100.4	0.4
Lake water	20	21.0	6.4	95.2	5.0
	40	38.9	4.1	102.8	3.0
	80	81.2	1.3	98.5	2.0
Tap water	20	21.3	6.4	93.9	6.5
	40	42.0	4.1	95.2	5.0
	80	79.2	1.3	101.0	1.0

*Average data of three replicates.

## Conclusion

In conclusion, we have developed a new NpI fluorescent chemodosimeter for the rapid detection of the Hg^2+^ ion. Dipeptidomimetic NpI displayed distinct turn-on fluorescence alteration toward Hg^2+^ in aqueous solution and can act as a highly sensitive sensor for the real-time analysis. Besides that, NpI exhibited fast fluorescence response and low detection limit of 14.2 nM. Furthermore, the probing mechanism of NpI was proposed as Hg^2+^-assisted hydration conversion of isocyano group to amino groups and was verified by NMR spectroscopy, MS analysis, and DFT calculation. It is worth mentioning that dipeptidomimetic NpI successfully penetrated the cell membrane and was efficiently used for fluorescence confocal bioimaging of Hg^2+^ ion in MCF-7 cells. Real water sample measurements further demonstrated the practical utility of this probe for environmental applications.

## Data Availability

The original contributions presented in the study are included in the article/Supplementary Material, further inquiries can be directed to the corresponding authors.
