# Supplementary material for: Design and Synthesis of Dipeptidomimetic Isocyanonaphthalene as Enhanced-Fluorescent Chemodosimeter for Sensing Mercury Ion and Living Cells
Source: Front Chem. 2022 Mar 4;10:813108. doi: 10.3389/fchem.2022.813108 (PMC8934403; doi:10.3389/fchem.2022.813108)
Supplement: Supplementary file 1 [file DataSheet1.docx]

***Supporting Information***

**Dipeptidomimetic isocyanonaphthalene as** **enhanced-fluorescent chemodosimeter for sensing mercury ion and living cells**

**Xiao-Juan Wang, Gao-Wei Li*, Yi-Peng Cheng, Qiu-Ling Sun, Yuan-Qiang Hao*, Chen-Hong Wang, Lan-Tao Liu**

*College of Chemistry and Chemical Engineering, Henan Engineering Laboratory of Green Synthesis for Pharmaceuticals, and Henan Key Laboratory of Biomolecular Recognition and Sensing, Shangqiu Normal University, Shangqiu, 476000, China*

*E-mail: ligaowei@sqnu.edu.cn (G.-W. Li), hao0736@163.com (Y.-Q. Hao)*

**Contents**

[1 Experimental procedures and characterization data 2](#_Toc93174491)

[1.1 Materials and instrumentation 2](#_Toc93174492)

[1.2 Cell culture and fluorescence imaging 3](#_Toc93174493)

[1.3 Synthesis and characterization of intermediates and sensor NpI 3](#_Toc93174494)

[2. NMR spectrum of compound 1 6](#_Toc93174495)

[3. NMR spectrum of compound 2 7](#_Toc93174496)

[4. NMR, IR and HRMS spectrum of sensor NpI 8](#_Toc93174497)

[5. Screening of buffer system for sensor NpI 10](#_Toc93174498)

[6. pH dependence of sensor NpI 10](#_Toc93174499)

[7. The emission spectra of intermediate 1, intermediate 1-Hg^2+^ and NpI-Hg^2+^ 11](#_Toc93174500)

[8. Detection mechanism by NMR and MS 11](#_Toc93174501)

[9. Comparison of fluorescent probes for Hg^2+^ 13](#_Toc93174502)

1 Experimental procedures and characterization data

## 1.1 Materials and instrumentation

**Solvents and reagents:** All starting materials, analytical reagents or solvents were obtained commercial suppliers and used as received without further purification unless otherwise denoted. Dried dichloromethane (DCM) was distilled from CaH_2_ under argon atmosphere. Triethylamine was distilled from KOH before use. Moisture and/or air sensitive experiments were conducted under argon atmosphere using typical Schlenk techniques. Analytical thin-layer chromatography was performed using silica gel 60 F254 plates.

The chlorate salts used in stock solutions of metal ions were Al^3+^, Co^2+^, Cd^2+^, Cr^3+^, Fe^3+^, Ca^2+^, K^+^, Mg^2+^, Li^+^, Hg^2+^ and Na^+^, other Cu^2+^, Mn^2+^, Ni^2+^, Pb^2+^, Sr^2+^ and Zn^2+^ were prepared from its sulfate salts. Concentrations of above-mentioned solutions were controlled at 20 ×10^-3^ M in deionized water and were diluted subsequently to different concentration stocks for next use.

The ^1^H NMR and ^13^C NMR spectra were measured on a Bruker Advance 400 MHz spectrometer with chemical shifts reported in ppm (in CDCl_3_ or DMSO-*d*_6_). FT-IR spectra were recorded on Perkin-Elmer Spectrum BX FT-IR system. LC-MS analysis was performedon an Agilent 6530 TOF LC/MS mass spectrometerwith a Poroshell 120 EC-C18 column (4.6×50 mm, 2.7 um). All pH measurements were made with a Model PHS-3C. UV-visible spectra were recorded on a Perkin-Elmer (Lambda 850) spectrometer. Fluorescence measurements were performed at room temperature on Agilent technologies carry eclipse fluorescence spectrophotometer. Melting points were determined using YRT-3 melting point apparatus and are uncorrected. Ultra-pure water was obtained by a Milli-Q Millipore filter system (Millipore Co., Ltd., Bedford, MA, USA) with a resistivity of 18.2 MΩ cm^−1^. All NMR experiments were carried out on a Bruker Avance 400 spectrometer using CDCl_3_ and DMSO-*d*_6_ as the solvents with tetramethylsilane as the internal standard.

Stock solution of **NpI** (DMSO, 2 mM) was prepared in a 5 mL volumetric flask. For optical testing, 10 μL solution of **NpI** was transferred to a 1 mL quartz colorimetric cuvette containing 1.98 mL PBS (10 mM, pH = 7.4) to give the sample solution. The final concentration of **NpI** in the sample solution was fixed at 1.0 μM throughout the absorption and fluorescence spectral measurement. All tests were measured at room temperature. Excitation wavelength was set as 350 nm and the excitation and launch slits were fixed as 5/5 nm throughout the fluorescence measurement. In the probing experiment, 1 μL of analytes (2.0 mM) was introduced to the test solution by pipette (the final concentration of analytes in probing medium was 1 μM) and incubated for 1 min before optical measurement. The Hg^2+^-induced time-dependent fluorescence alteration was performed under the identical condition with different incubating time. The concentration-dependent fluorescence alteration of **NpI** was recorded with different concentration of Hg^2+^.

## 1.2 Cell culture and fluorescence imaging

Confocal laser scanning microscopy was used and the blue channel was employed for fluorescence imaging. MCF-7 cells (human malignant melanoma cells) were seeded overnight to adhere on glass-bottomed dish in DMEM culture medium in air containing 5% CO_2_ at 37 °C. The control group of MCF-7 cells were stained with probe **NpI** (10.0 µM) for 30 min, and mounted on the microscope stage to capture the cellular fluorescence images using Zeiss LSM710 microscope. The experiment group of MCF-7 cells were first incubated with **NpI** (10.0 µM) for 30 min and then dealed with HgCl_2_ (50 μM) for 30 min. After the above breeding experiment, cells were rinsed with PBS buffer and imaged on confocal microscope immediately.

## 1.3 Synthesis and characterization of intermediates and sensor NpI

**Scheme S1.** The synthesis of sensor **NpI**

*Synthesis of dipeptidyl amide* ***1***

*L*-Valanine benzyl ester hydrochloride (1.3 g, 6 mmol) was dissolved in 20 mL THF and cooled to 0 °C, distilled triethylamine (2.6 mL, 18.2 mmol) was added dropwise, and the resulting mixture was stirred for 0.5 h. Subsequently, 6-amino-2-naphthoic acid (1.13 g, 6 mmol), 1-hydroxybenzotriazole (HOBt, 0.09 g, 0.6 mmol), and *N*,*N*-dicyclohexylcarbodiimide (DCC, 1.5 g, 7 mmol) were added. The reaction suspension was then stirred at ambient temperature for 12 h. The precipitate formed byproduct (dicyclohexyl urea) was filtered off and the filtrate was concentrated under reduced pressure. The white targate product was brought into ethyl acetate, the crude purple product was washed with brine water, the organic layer was collected and dried over anhydrous Na_2_SO_4_, and concentrated to dryness. The residues were purified by silica gel column chromatography using petroleum ether-ethyl acetate as eluent to afford an pink solid (1.9 g, yield: 84%). M. p. = 131.1-131.6 °C. [α]20 D = +51.13 (*c* 0.20, in CHCl_3_). ^1^H NMR (400 MHz, CDCl_3_) *δ* (ppm) 8.15 (d, *J* = 1.9 Hz, 1H), 7.74- 7.71 (m, 2H), 7.60 (d, *J* = 8.6 Hz, 1H), 7.47 – 7.29 (m, 5H), 7.03 (d, *J* = 8.7 Hz, 2H), 6.75 (d, *J* = 8.6 Hz, 1H), 5.25 (d, *J* = 12.2 Hz, 1H), 5.18 (d, *J* = 12.2 Hz, 1H), 4.88 (dd, *J* = 8.6, 4.8 Hz, 1H), 2.38 – 2.27 (m, 1H), 1.01 (d, *J* = 6.9 Hz, 3H), 0.97 (d, *J* = 6.8 Hz, 3H). ^13^C NMR (100 MHz, CDCl_3_) *δ* (ppm) 172.3, 167.5, 146.1, 136.8, 135.4, 130.5, 128.6, 128.5, 128.4, 127.7, 126.1, 124.2, 118.9, 107.9, 67.2, 57.4, 33.9, 31.8, 19.1, 17.9. HRMS m/z (ESI) anal. calcd for C_23_H_24_N_2_O_3_ [M+Na] ^+^ 399.1787, found 399.1749.

*Synthesis of N-formyl-dipeptidyl amide* ***2***

The amino formylation reaction of dipeptidyl amide **1** in a mixture of formic acid (FA) and acetic anhydride. After a one-pot mixture of FA (14.12 mL, 10.2 mmol) and acetic anhydride (0.76 mL, 8.04 mmol) was stirred at ambient temperature for 2 h. Dipeptidyl amide **1** (1.8 g, 4.79 mmol) in ethyl acetate (20 mL) was added to the resulting mixture. The dispersion solution was stirred 48 h. To the solution was added a lot of ethyl acetate (50 mL), the organic phase was washed two times with 50 mL brine, and then dried over anhydrous Na_2_SO_4_ and filtered. After the solvent was removed under reduced pressure, The residues were purified by silica gel column chromatography using petroleum ether -ethyl acetate (1:1, v/v) as eluent to afford an orange solid (1.4 g, yield: 72%). M. p. = 147.5– 148.1 °C. [α]20 D = +49.91 (*c* 0.20, in CHCl_3_). ^1^H NMR (400 MHz, DMSO-*d6*) rotamer, ^1^H NMR (400 MHz, DMSO) *δ* (ppm) 10.52 (s, 1H, CHO), 10.52 (s, 0.4H, NH), 9.01 (s, 1H), 8.48 – 8.33 (m, 2H), 8.01 (d, *J* = 8.9 Hz, 1H), 7.95 – 7.78 (m, 2H), 7.64 (d, *J* = 8.9 Hz, 1H), 7.40 – 7.37 (m, 5H), 5.20 (d, *J* = 12.5 Hz, 1H), 5.15 (d, *J* = 12.6 Hz, 1H), 4.38 (t, *J* = 7.6 Hz, 1H), 2.26 – 2.19 (m, 1H), 1.01 (d, *J* = 6.7 Hz, 3H), 0.95 (d, *J* = 6.8 Hz, 3H). ^13^C NMR (100 MHz, DMSO-*d6*) *δ* (ppm) 172.1, 167.6, 160.5, 137.8, 136.4, 135.3, 130.4, 130.3, 129.2, 128.8, 128.5, 128.4, 128.2, 127.7, 125.6, 120.8, 115.5, 66.31, 59.3, 30.0, 19.6, 19.5. HRMS m/z (ESI) anal. calcd for C_24_H_24_N_2_O_4_ [M+Na] ^+^ 427.1736, found 427.1645.

*Synthesis of probe* ***NpI***

Under the argon atmosphere, *N*-formyl-dipeptidyl amide 2 (0.85 g, 2.1 mmol) was dissolved in 25 mL dry CH_2_Cl_2_, triethylamine (Et_3_N, 0.75 mL, 5.5 mmol, 2.0 equiv) was added, and the reaction mixture was cooled to 0 ºC. Over a period of 1 h, a solution of triphosgene (BTC, 443 mg, 1.5 mmol) in 10 mL dry CH_2_Cl_2_ was added dropwise into the mixture via additional vessel, the temperature of reaction system was maintained at 0 ºC. The resulting reaction mixture was stirred for an additional 3 h at room temperature. An ice-cold saturated NaHCO_3_ aqueous solution (10 mL) was added and the mixture was stirred vigorously for 5 min. The organic part was separated and next extracted with CH_2_Cl_2_, washing once with brine and dried over anhydrous Na_2_SO_4_, filtered, and concentrated. Purification on silica column chromatography (Petroleum ether: EtOAc = 3:1) afforded desired probe NpI (0.61 g, yield: 81%). M. p. = 128.1– 128.5 °C. [α]20 D = +47.48 (*c* 0.20, in CHCl_3_). ^1^H NMR (400 MHz, CDCl_3_) *δ* (ppm) 8.30 (d, *J* = 1.7 Hz, 1H), 7.94 – 7.86 (m, 4H), 7.47 (d, *J* = 8.7 Hz, 1H), 7.43 – 7.30 (m, 5H), 6.87 (d, *J* = 8.6 Hz, 1H), 5.27 (d, *J* = 12.2 Hz, 1H), 5.20 (d, *J* = 12.2 Hz, 1H), 4.89 (dd, *J* = 8.6, 4.8 Hz, 1H), 2.37- 2.32 (m, 1H), 1.02 (d, *J* = 6.9 Hz, 3H), 0.98 (d, *J* = 6.8 Hz, 3H). ^13^C NMR (100 MHz, CDCl_3_) *δ* (ppm) 172.1, 166.7, 165.5, 135.2, 134.2, 133.2, 132.1, 130.8, 128.8, 128.7, 128.6, 128.5, 128.3, 127.5, 125.6, 125.4, 124.4, 67.4, 57.6, 31.8, 19.1, 17.9. FT-IR (cm^−1^): 3265, 2950, 2125, 1730, 1635, 1535, 1200, 700. HRMS (ESI): m/z: anal. calcd for C_24_H_22_N_2_O_3_ [M+Na] ^+^ 409.1630, found 409.1649.

2. NMR spectrum of compound 1

**Fig. S1.** ^1^H NMR (400 MHz, CDCl_3_) spectrum of compound **1**.

**Fig. S2.** ^13^C NMR (100 MHz, CDCl_3_) spectrum of compound **1**.

3. NMR spectrum of compound 2

**Fig. S3**. ^1^H NMR (400 MHz, CDCl_3_) spectrum of compound **2**.

**Fig. S4**. ^13^C NMR (100 MHz, CDCl_3_) spectrum of compound **2**.

4. NMR, IR and HRMS spectrum of sensor NpI

**Fig. S5**. ^1^H NMR (400 MHz, CDCl_3_) spectrum of sensor **NpI**.

**Fig. S6**. ^13^C NMR (100 MHz, CDCl_3_) spectrum of sensor **NpI**.

**Fig. S7**. DEPT 135 ^13^C NMR (100 MHz, CDCl_3_) spectrum of sensor **NpI**.


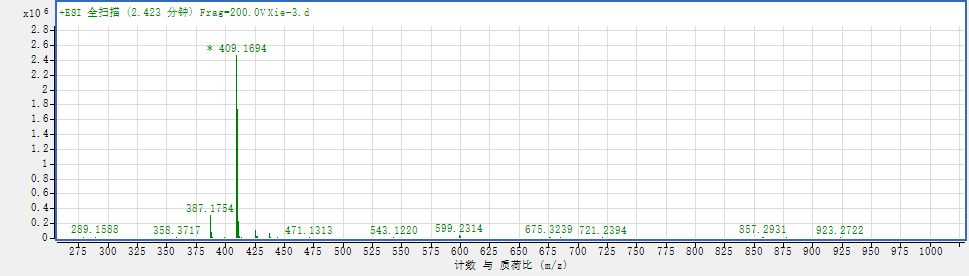


**Fig. S8**. HRMS (ESI) spectrum of sensor **NpI**.


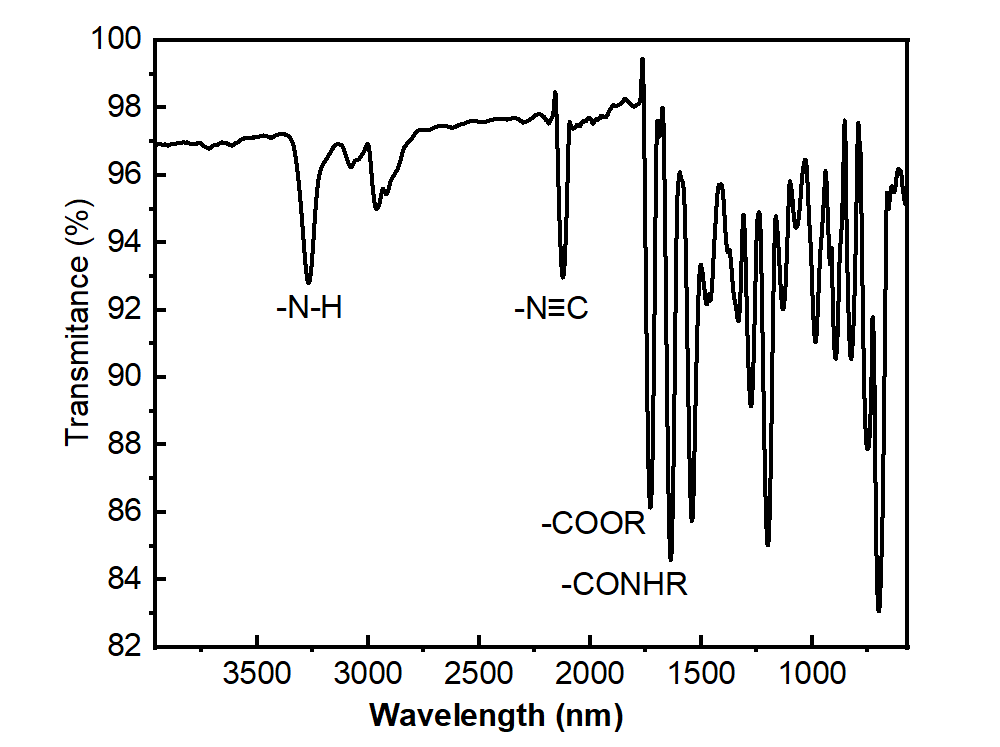


**Fig. S9**. FT-IR spectrum of sensor **NpI**.

5. Screening of buffer system for sensor NpI


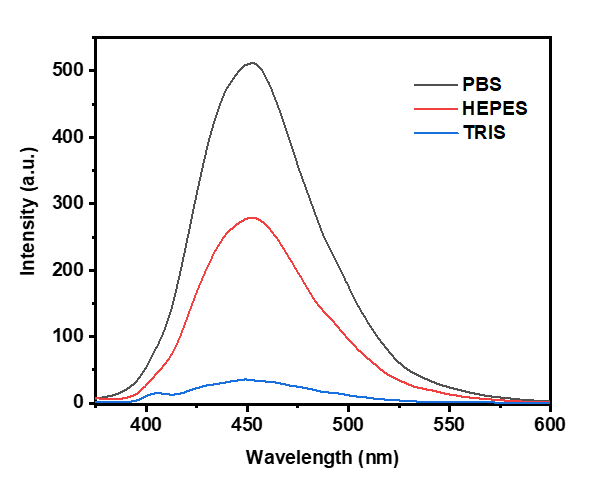


**Fig. S10**. Screening of buffer system for the sensing application spectrum of sensor **NpI**. Excitation wavelength is 350 nm.

6. pH dependence of sensor NpI


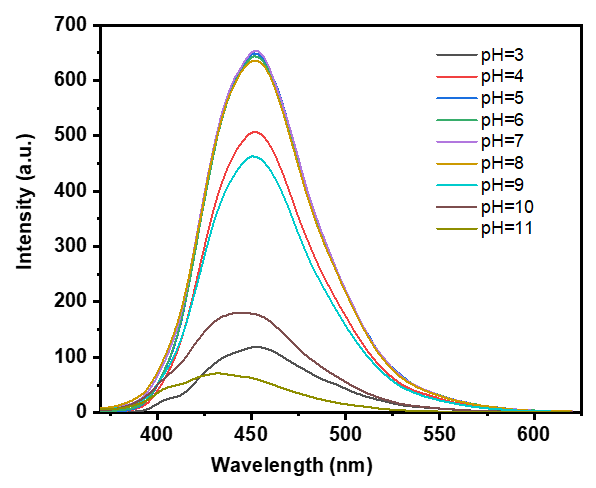


**Fig. S11**. Influence of pH on the fluorescence probing of Hg^2+^ by sensor **NpI**.

7. The emission spectra of intermediate 1, intermediate 1-Hg^2+^ and NpI-Hg^2+^


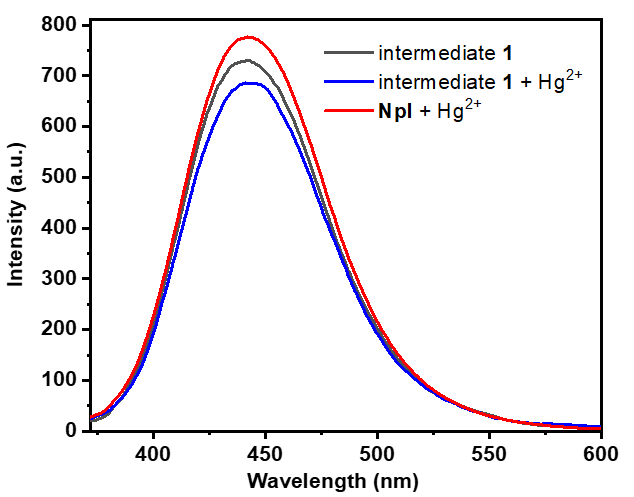


**Fig. S12**. Fluorescence spectra of intermediate **1**, intermediate **1**-Hg^2+^ and **NpI**-Hg^2+^ in PBS aqueous (pH =7.4, containing 0.5% DMSO).

8. Detection mechanism by NMR and MS

**Fig. S13.** Evolution of the ^1^H NMR spectra of **NpI** with addition of 1.0 equiv Hg^2+^ in the CDCl_3_ solution


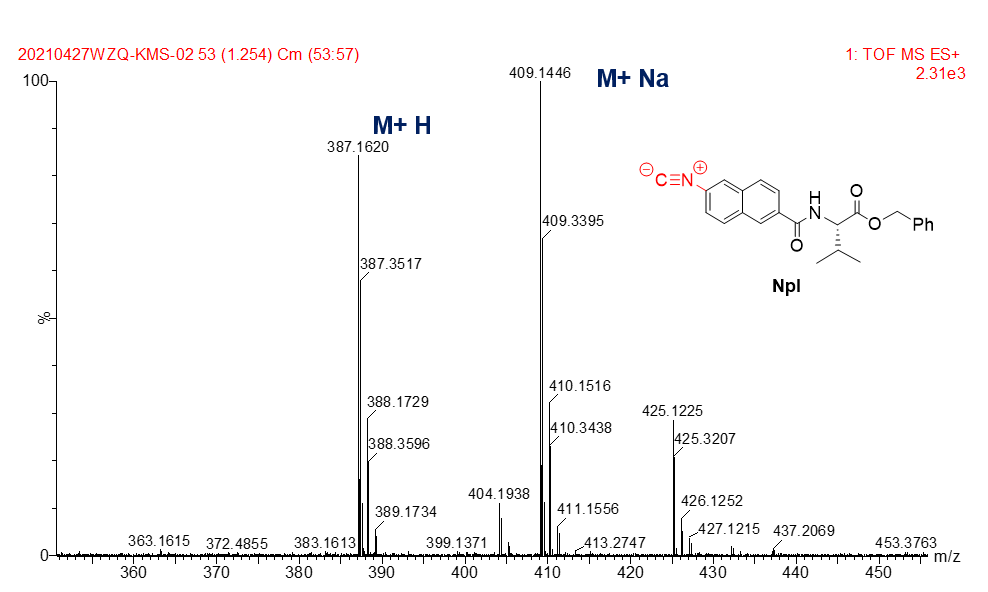


**Fig. S14**. MicroTOF-Q type Qq-TOF MS spectrum of **NpI**.


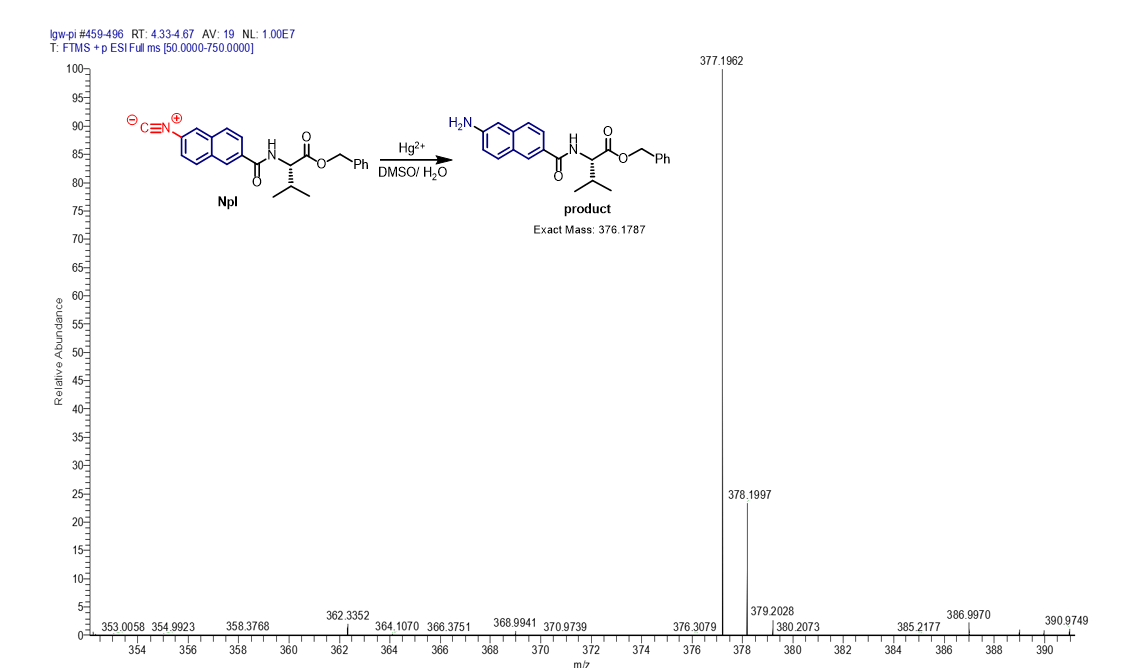


**Fig. S15**. MicroTOF-Q type Qq-TOF MS spectrum of the reaction mixture obtained in the reaction of **NpI** with the mixture of water/DMSO and HgCl_2_ after 5 min.


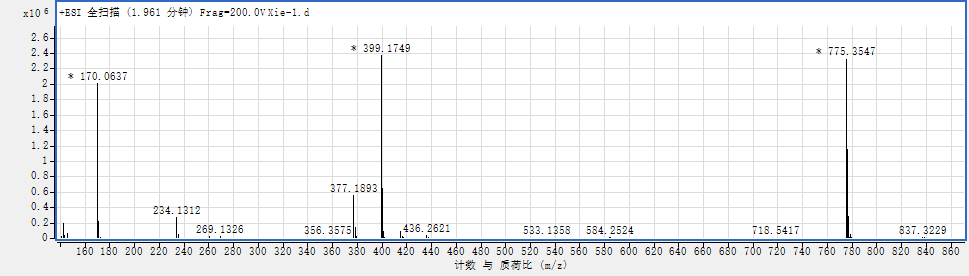


**Fig. S16**. HRMS (ESI+) spectrum of intermediate **1**.

9. Comparison of fluorescent probes for Hg^2+^

**Table S1.** A comparative study of the present probe with selected previously reported Hg^2+^

| **Probes** | **Response mode** | **Limit of detection** | **Response time** | **Reference** |
| --- | --- | --- | --- | --- |
|  | Turn on | 164 nM | 60 min | J. Photoch. Photobio. A. 2020, 387, 112160. |
|  | Turn off | 90 nM | 30 min | J. Photochem. Photobiol. Chem. 2018, 364, 503-509. |
|  | Colourimetric | 56.3 nM | - | RSC Adv. 2018, 8, 39854-39864 |
|  | Turn on | 0.63 μM | - | J. Lumin. **2019,** 208, 519-526 |
|  | Turn on | 0.87 μM | 1 min | Sens. Acutators B Chem. 2017, 245, 462-469 |
|  | Turn off | 143 nM | - | J. Mol. Liq. 2017, 248, 668-677 |
|  | Turn on | 0.12 μM | 30 min | Sens. Acutators B Chem. 2017, 243, 678-683 |
|  | Turn off-on | 0.18 μM | 5 min | Sens. Acutators B Chem. 2017, 249, 217-228 |
|  | Ratiometric | 50 nM | 5 min | Org. Lett. 2010, 12, 5310-5313 |
|  | Ratiometric | 0.7 μM | 30 min | Spectrochimica Acta Part A 2018, 201, 54-60 |
|  | Ratiometric | 6 nM | 1 min | Talanta, 2019, 201, 165-173 |
|  | Ratiometric | 91 nM | 1.5 min | Sensors actuat B Chem.  2018, 255, 3074-3084. |
|  | Turn on | 14.2 nM | within 1 min | This work |
